# MeSCoT: the tool for quantitative trait simulation through the mechanistic modeling of genes’ regulatory interactions

**DOI:** 10.1093/g3journal/jkab133

**Published:** 2021-04-27

**Authors:** Viktor Milkevych, Emre Karaman, Goutam Sahana, Luc Janss, Zexi Cai, Mogens Sandø Lund

**Affiliations:** Center for Quantitative Genetics and Genomics, Aarhus University, Blichers Allé 20, 8830 Tjele, Denmark

**Keywords:** genomic regulatory network, genomic architecture, complex trait, epistasis, omnigenic model

## Abstract

This work represents a novel mechanistic approach to simulate and study genomic networks with accompanying regulatory interactions and complex mechanisms of quantitative trait formation. The approach implemented in MeSCoT software is conceptually based on the omnigenic genetic model of quantitative (complex) trait, and closely imitates the basic *in vivo* mechanisms of quantitative trait realization. The software provides a framework to study molecular mechanisms of gene-by-gene and gene-by-environment interactions underlying quantitative trait’s realization and allows detailed mechanistic studies of impact of genetic and phenotypic variance on gene regulation. MeSCoT performs a detailed simulation of genes’ regulatory interactions for variable genomic architectures and generates complete set of transcriptional and translational data together with simulated quantitative trait values. Such data provide opportunities to study, for example, verification of novel statistical methods aiming to integrate intermediate phenotypes together with final phenotype in quantitative genetic analyses or to investigate novel approaches for exploiting gene-by-gene and gene-by-environment interactions.

## Introduction

Genome-wide single nucleotide polymorphisms (SNPs) have been used in animals and plants to map genes for many traits, leading to discovery of the causal genes and mutations for several mendelian traits but rarely for quantitative (complex) traits, those represent majority of traits that are of economic importance in agriculture (Goddard and Hayes 2009). Genetic variation in quantitative traits is considered to be determined by a large number of loci with small to moderate effects, which are individually undetectable by genome-wide association studies (GWAS) due to limited sample size and stringent genome-wide significance threshold. Although genomic prediction (Meuwissen *et al.* 2001) is fundamentally different from GWAS in that it involves the use of all SNPs regardless of their statistical significance, a better understanding of the genetic architecture that underlies quantitative traits could improve the predictive ability of models (Suravajhala *et al.* 2016; Fang *et al*. 2017 ).

The trait-specific marker maps are normally regarded as genomic architecture of a trait (Flint and Mackay 2009; Hayes *et al.* 2010). However, recent discoveries suggest that an intricate gene networks with accompanying regulatory interactions constitute genetic architecture of quantitative trait and, therefore, responsible for complex phenotype formation (Boyle *et al.* 2017; Liu *et al.* 2019b; Chateigner *et al.* 2020). Hence, marker maps alone cannot guarantee accurate polygenic predictions without accounting for underlying genetic regulatory networks with related nonadditive genetic interactions (Dai *et al.* 2020).

Nonlinear interactions between segregating loci as a natural consequence of existence of genomic regulatory networks, known as epistasis, is a common feature of genetic architecture of quantitative trait (Mackay 2014). Continuous discussion of a role and importance of epistasis, which has been initiated several decades ago (Cockerham 1954; Kojima 1959), is persistently under active debate these days (Hill *et al.* 2008; Mäki-Tanila and Hill 2014; Huang and Mackay 2016; Ehrenreich 2017; Dai *et al.* 2020; Duenk *et al.* 2020). Such interest, viewed in context of quantitative genetics in general and genomic prediction in particular, creates a constant demand for tools to simulate realistic phenotypic data derived from known genomic architecture with multilocus interactions.

Mapping gene interactions *in vivo* is challenging task (Mackay 2014; Ehrenreich 2017). This makes *in silico* generated expression data widely accepted (Sargolzaei and Schenkel 2009; Faux *et al.* 2016; Angelin-Bonnet *et al.* 2019; Liu *et al.* 2019a). Though, mRNA and protein concentrations form a molecular trait (Claringbould *et al.* 2017; Angelin-Bonnet *et al.* 2019), it is not sufficient to consider this as a complex trait in the sense of quantitative genetics.

A common approach to simulate a complex (quantitative) trait is sampling gene-by-gene (G×G) interaction effects for some arbitrary pairwise markers, which gives genotypic values. Sampling additional “error” effects that imitate gene-by-environment (G×E) interactions simulate phenotypic values (Forneris *et al.* 2017; Vitezica *et al.* 2017; Momen *et al.* 2018; Wang *et al.* 2019; Dai *et al.* 2020; Duenk *et al.* 2020). Unfortunately, such approach ignores nonrandom genes co-regulation within a genomic network and rather allows imitation of extra variance in data due to randomly generated interactions based on genomic maps than build phenotypic values using full genomic architecture.

Here, we present a novel mechanistic approach to *in silico* quantitative trait simulation implemented within MeSCoT (Mechanistic Simulation of Complex Trait) software. The approach realizes core *in vivo* mechanisms of complex trait formation and quantitatively maps genotypic and phenotypic variation into the molecular mechanisms of gene expression. Therefore, it constitutes a computational framework for G×G and G×E interaction studies as well as for verification of novel and existing statistical methods in quantitative genetics.

Primarily, the software performs a detailed mechanistic simulation of gene regulatory interactions for variable genomic architectures and generates transcriptional/translational genes’ products data. Basically, such MeSCoT functionality overlaps with some other software solutions, which have been proposed in the last decades, see the detailed overview of principal algorithms and key features in Angelin-Bonnet *et al.* (2019). However, besides the detailed mechanistic model of gene regulatory interactions, the major contribution of our approach is due to the novel SNP and omnigenic genetic models implemented within the MeSCoT software. These models allow detailed mechanistic studies of impact of genetic and phenotypic variance on gene regulation and, hence, help to reveal molecular mechanisms linking the heritability and variation in molecular traits.

## Methods

### Model

The underlying conceptual model for our simulation framework is an *omnigenic* model of quantitative trait architecture proposed in Boyle *et al.* (2017) and Liu *et al.* (2019b). According to this model, complex formation of quantitative trait is due to direct genetic contributions from core genes and indirect contributions from peripheral genes. While core genes affect trait explicitly, peripheral genes contribute to trait only through *trans*-regulatory effects on core genes. In case of complex genomic architectures, where the core genes are normally co-regulated, peripheral genomic variation is magnified such that most of variance is driven by weak *trans*-effects. Therefore, such effects are responsible for most of trait heritability. While products of core genes are responsible for direct quantitative trait formation, many peripheral gene variants determine cumulative polygenic effect.

Besides the *omnigenic* model, we consider a number of additional assumptions. All genes in a network are subject to an explicit transcriptional regulation where production rate of gene’s mRNA is proportional to a binding probability of RNA polymerase II complex (RNAP II). The binding probability of RNAP II is mediated by the products of other genes from the same network and is modeled here using a widely accepted statistical thermodynamic approach (Ackers *et al.* 1982; Shea and Ackers 1985; Bintu *et al.* 2005; Chu *et al.* 2009). We do not consider a direct regulation of mRNA translation but rather model production rate of gene’s products as a linear function of mRNA concentration.

The following matrix equation represents a mathematical formulation of a model of genes regulatory interactions:

(1) $$\dot{c}=\mathrm{Kb}-\mathrm{Zc}+\mathrm{Qc},$$

where c and b are vectors of variables expressed as

$$c=e_{1}⊗x(t)+e_{2}⊗s(t);$$

$$b=e_{1}⊗p(s)+e_{2}⊗x(t-\tau );$$

$$K=\left[\begin{array}{cc} K_{x} & \\ & K_{s} \end{array}\right],Z=\left[\begin{array}{cc} Z_{x} & \\ & Z_{s} \end{array}\right];$$

$e_{1},\;e_{2}$ are basis vectors in (2D real vector space) $R^{2}$; t is time; x is a vector of mRNA concentrations; s is a vector of protein concentrations; K and Z are the diagonal matrices of rate and degradation constants respectively; p is a vector of binding probabilities of RNAP II to a promoter region of a gene; τ is time delay due to a molecular diffusion (Zhang *et al.* 2012; Chaplain *et al.* 2015; Macnamara *et al.* 2019); Q is stochastic diagonal matrix with elements $\gamma q_{ii}(t)$, where $q_{ii}(t)∼N(0,{\sigma_{q}}^{2})$, ${\sigma_{q}}^{2}\ll 1$ is a variance and γ is constant; and the upper dot $\left(\dot{}\right)$ indicates time derivative; ⊗ is the Kronecker product.

Binding probability of RNAP II to gene’s promoter

(2) $$p\left(s\right)=2{\left[I+{F\left(s\right)}^{-1}\;\text{exp}\;\left(-G_{p}\right)H\right]}^{-1}\;1_{n},$$

where I is n×n identity matrix; F(s) is n×n diagonal matrix of a gene’s regulatory factors where ${F(s)}_{ii}=\det({{F(s)}_{A}}_{i}{{F(s)}_{R}}_{i})$; $G_{p}$ is n×n diagonal matrix of a relative free energies related to a gene’s RNAP II binding; H is n×n diagonal matrix of RNAP II-binding constants; $1_{n}$ is n×1 vector where all elements are one; n is a number of genes in the network.

Gene’s regulatory factors

(3) $${{F(s)}_{A}}_{i}={\left[h_{A}I+{S_{\mathrm{Ai}}}^{2}\;\text{exp}\;(-G_{A})\Phi \right]}^{1/n}{\left[h_{A}I+{S_{\mathrm{Ai}}}^{2}\;\text{exp}\;(-G_{A})\right]}^{-1/n},$$

(4) $${{F(s)}_{R}}_{i}={\left[I+h_{R}{S_{\mathrm{Ri}}}^{2}\;\text{exp}\;(-G_{R})\right]}^{-1/n},$$

where ${{F(s)}_{A}}_{i}$ and ${{F(s)}_{R}}_{i}$ are n×n diagonal matrices of gene’s activator and repressor factors, respectively; $S_{\mathrm{Ai}}$ ($S_{\mathrm{Ri}}$) is n×n diagonal matrix of concentrations of activators (repressors) molecules for a gene i; $G_{A}$ ($G_{R}$) is n×n diagonal matrix of a relative free energies related to a gene’s activators (repressors) binding; Φ is n×n diagonal matrix of activators binding interaction constants; $h_{A}$ ($h_{R}$) is activators (repressors) constant.

A network geometry accounted in the model through the equations for $S_{\mathrm{Ai}}$ and $S_{\mathrm{Ri}}$

(5) $$S_{\mathrm{Ai}}=\mathrm{diag}(\mathrm{diag}\left(s\left(t-\tau \right)\right)\;Ae_{i}),$$

(6) $$S_{\mathrm{Ri}}=\mathrm{diag}(\mathrm{diag}\left(s\left(t-\tau \right)\right)\;Re_{i}),$$

where $\mathrm{diag}:\;R^{n}\to R^{n\times n}$ is operator which transforms n×1 vector to n×n diagonal matrix, $\mathrm{diag}\left(s\right)=\sum_{j}^{n}e_{j}^{T}se_{j}e_{j}^{T}$; $e_{j}$ and $e_{i}$ are the *j*-th and *i*-th basis vectors in $R^{n},$ respectively; **A**, **R** are the adjacency matrices of activators and repressors subnetworks, respectively, so the adjacency matrix of the genomic network is N=A+R.

Regulators subnetworks are deduced at the initial stage of simulation process either from *in vivo* inferred genomic network or from *in silico* generated networks. Whereas *in vivo* network comes from an external source, a synthetic network geometry can be generated in place.

We model trait as a superposition of weighted core gene products

(7) $$y=\left[W⊙\bar{S}\right]\;1_{m},$$

where y is m×1 vector of trait values; m is a number of individuals in population; W is $m\times n_{c}$ matrix of weights; $n_{c}$ is a number of core genes; $\bar{S}$ is $m\times n_{c}$ matrix of time averaged and normalized values of core genes’ proteins; ${\bar{S}}_{ji}={\bar{s}}_{ji}/{\bar{s}}_{ri}$, where ${\bar{s}}_{ji}$ is time-averaged solution of the model for a gene *i* in individual j, ${\bar{s}}_{ri}$ is time-averaged solution for a gene *i* in reference genotype; $1_{m}$ is m×1 vector where all elements are one; ⊙ is Hadamard product.

### Accounting for polymorphism and genomic variation

In order to ensure a quantitative diversity in functioning of genetic regulatory mechanisms across genome and guarantee genomic variation in population, we sample model parameters responsible for transcription from the normal distribution and adjust to relative markers’ effects (mapping genomic polymorphism on a molecular level of gene expression).

Recall $K_{x}\in R^{n\times n}$ a diagonal parameter matrix that represents specific to genotype j values of genes’ expression rates

$${K_{x}}_{j}=\mathrm{diag}\left(\;PM^{T}e_{j}\;\right),$$

where $P∼N\left(P_{\mu },\Sigma_{P}\right)$ is diagonal matrix of expression rates, $P_{\mu }$ is a mean expression rate, that is determined through the software input interface; $\Sigma_{P}$ is a variance of expression rate, accepted here as $\Sigma_{P}=\kappa \cdot P_{\mu }$, where κ (Eq.8) is a response parameter determined through the software interface; M is m×n matrix of relative markers’ effects; $e_{j}$ is m×1 unit vector with one in the *j*-th position and zeros elsewhere, m is a number of individuals in population.

P has to be sampled once (hence, is the same across all genotypes) and parametrically determines an expression variability due to existence of different functional classes of genes in the network (simply saying, all genes are different in terms of the expression rates). $M^{T}e_{j}$ is calculated for each genotype and realizes variation in gene expression due to genomic polymorphism.

(8) $$M=\frac{\kappa }{3}\left(M_{\mathrm{pop}}-M_{\mathrm{ref}}\right)+1,$$

where κ∈[0,1] is a G×G response parameter; $M_{\mathrm{pop}}$ is a genotypic matrix that contains which marker alleles each individual inherited; $M_{\mathrm{ref}}$ is a matrix of reference genotypes; 1 is m×n matrix where all elements are one. Here, the reference genotype is a genotype that consists of markers’ variants with highest frequencies in population. There is one reference genotype per population, therefore, all rows in $M_{\mathrm{ref}}$ are the same.

### The model of G × E interaction

Besides the basic simulation, where G×G interactions are highlighted, the MeSCoT allows G×E studies by employing the following model

$$\mathrm{Env}∼N(0,{\sigma_{\mathrm{Env}}}^{2}),$$

$$K_{x}∼N(\mu_{x},\varphi {\mu_{x}}^{2}),$$

$$\varphi ={\sigma_{\mu_{x}}}^{2}/{\mu_{x}}^{2},$$

where Env is a virtual environment with variance ${\sigma_{\mathrm{Env}}}^{2}$; $K_{x}$ is the same as in Eq. 1; $\mu_{x}$ is an expectation of $K_{x}$; φ is a G×E response parameter, it is related to the environment as $\varphi ∼\omega {\sigma_{\mathrm{Env}}}^{2}$ where ω is a proportionality coefficient. Thus G×E interaction implemented on a genome’s molecular level realized in changes of mRNA transcription rates as a result of the environmental stress imitated by the response parameter φ.

### Network geometry

To generate scale-free genomic network with complex predictable geometry [such as modularity, network motifs, etc. (Newman 2006)], we simulate nonequilibrium dynamical evolution process of *d*-dimensional simplexes, which are fully connected graphs of (d+1) nodes (Bianconi and Rahmede 2016). First, we construct three different basal (correspondent to d=1,2,3) network geometries possessed known structural properties resembled real gene networks (Balaji *et al.* 2006). Here, we use Bianconi–Rahmede model (Bianconi and Rahmede 2016).

At this stage of network simulation, the software uses three sets of parameters (with two distinct parameters in each set that are defined through the input interface) dedicated to basal geometries: (1) the proportion of genes in each basal network; and (2) the configuration parameters that determine a shape of basal network.

The generated basal geometries then merged to form higher-order organizational structures. Merging is performed by iteratively establishing new edges between random nodes of basal geometries (graphs). The number of new edges is proportional to an order of the resulting graph and a number of merging iterations is determined through the input interface.

The resulting (merged) network represents geometrical model of quantitative trait architecture further used by the genes interaction model.

### Simulation workflow

**Figure 1:**
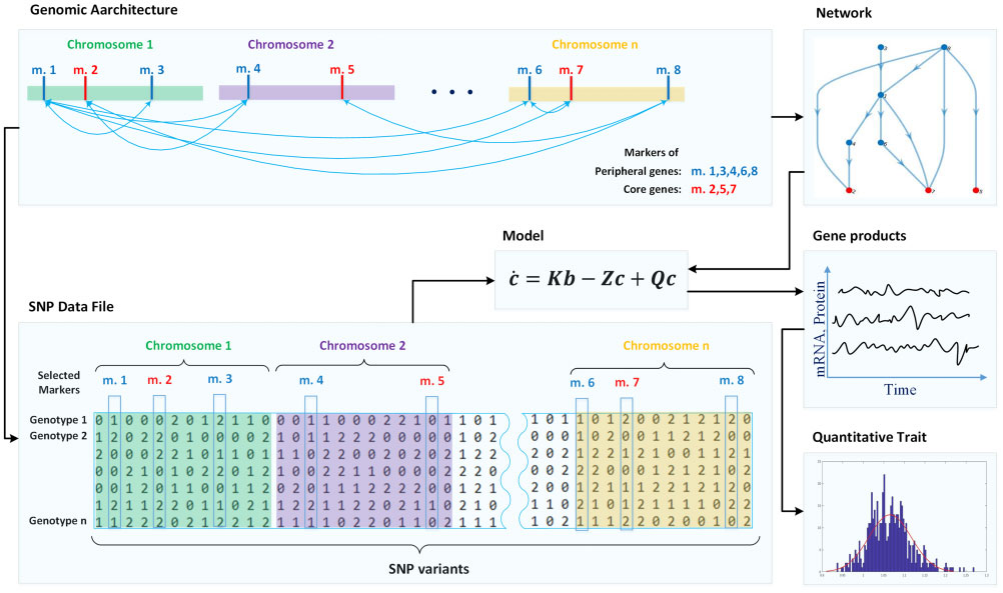
The schematic overview of MeSCoT software simulation workflow. The shaded areas depict distinct software workflow components: data blocks and functional units. The black arrows represent information flows within existing interfaces among the components. The “Genomic Architecture” is a data block of prior information regarding a modeled complex trait, such as peripheral and core genes (markers colored in blue and red, respectively), their locations and network relations (blue arrows). The prior information is used to build a (1) data file consisting the combined information for all genotypes (SNP variants) in population, the shaded area named “SNP Data File”; and (2) adjacency matrices for the modeled genomic network, the area named “Network.” The computational unit (“Model”) utilizes the genomic and network information to produce “Gene products” data that can be further used for the trait calculations, depicted within “Quantitative Trait” area.

The schematic overview of MeSCoT simulation workflow and a main input data structure is depicted in Figure 1. The *SNP data file* formed and structured according to assumed *Genomic Architecture* (prior information), which is also the source for *Network* construction. The *Model* utilizes genomic information and a network geometry and produce *Genes’ products* data (a time-series data of mRNA and protein concentrations for each gene in the network). The subset of time-averaged core genes’ products data forms an input information for the *Quantitative trait* model that produces a final output of simulated trait.

### The software interface and the data used for the case studies

**Figure 2:**
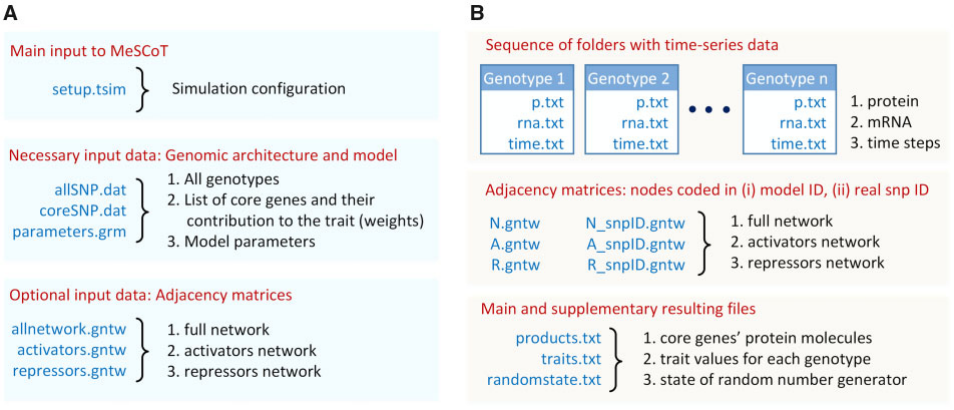
MeSCoT interface. (A) Input interface. (B) Output interface. Blue color indicates different types of files and folders; black color describes a function and purpose of files; red color marks different interface groups.

For the case studies, we shall consider a trait with a pure artificial genomic architecture determined by *in silico* generated genomic network. To this end, we generated genotypic data for 5000 individuals completely at random, by sampling allele counts from 0, 1, and 2, for 50 SNP loci (“allSNP.dat” file in Figure 2A). Among the 50 SNPs simulated, 12 were considered as being associated with core genes with equal contribution to the trait (“coreSNP.dat” file in Figure 2A). That is, the same weight was assigned to each gene product. The rest of the SNPs were considered as (associated with) peripheral genes (note, there is no special data file is required).

Figure 2 shows the MeSCoT interface and represents a grouping of different input files required for launching simulations (Figure 2A), as well as an output data supplied with successful completion of a particular simulation, Figure 2B.

### Numerical solution and model parameters

The model, which appeared as a system of stochastic differential equations with time delay (Equation 1), numerically reduced to two distinct problems: (1) if the stochastic matrix Q is determined to be a nonzero, we consider a pure stochastic problem where τ=0; otherwise (2) it is a time delay problem.

In the case of stochastic problem, the Euler–Maruyama method (Bayram *et al.* 2018) is used for numerical approximation. Here, we use a constant diffusion coefficient and standard Wiener process is parameterized via an input interface (a parameter that define variance of normal distribution while mean is always zero, see the Appendix 2 for the corresponding software keywords). A time step is determined as $\Delta t=5\cdot 10^{-4}\;T_{\max}$, where $T_{\max}$ is a maximum simulation time. The solution to the problem was implemented using SDE Toolbox (Picchini 2007).

In the case of time-delay problem, the extension of Runge–Kutta method is used for integration of time delay differential equations (Shampine and Thompson 2001). The initial step size is based on the slope of the solution at the initial time. The upper bound of step size is not fixed and is adjusted during the numerical integration. The solution to the problem was implemented using the MATLAB *dde23* solver.

Regardless of the numerical problem, the integration time span is defined through the input interface. A vector of initial value is determined as $1_{n}$.

To provide a greater flexibility of the approach implemented within MeSCoT, all adjustable model parameters (except one, which is predefined constant) can be determined through the software input interface (Appendix 2). Besides the possibility of direct parameters input, there are the default values for the number of parameters that can be used to configure simulations. The default values for the rates parameters are based on the results represented in Hausser *et al.* (2019).

### Data availability

The software was coded using MATLAB programming language and compiled to stand-alone executable, which does not require MATLAB environment to run the application. The executables (for Linux and Windows platforms) as well as the necessary documentation and examples are freely accessible via the MeSCoT supporting web site: https://genetics.ghpc.au.dk/vimi/mescot.

## Results and discussion

### Genomic network

**Figure 3:**
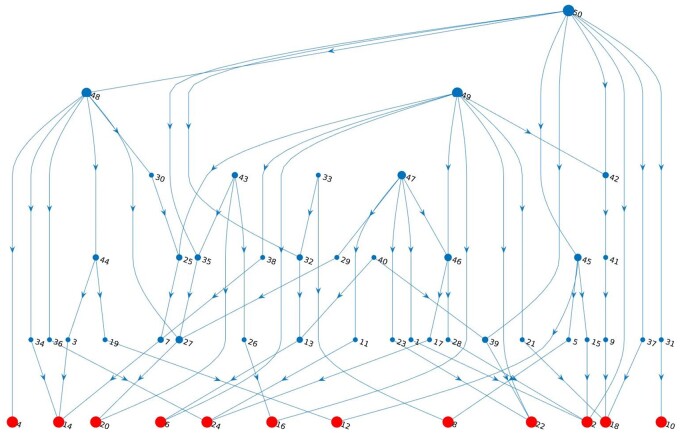
Simulated genomic network. Gene network geometry (directed graph) consisted of 50 genes among which 12 are core; the size of blue dots is proportional to the graph’s nodes degrees; red dots indicate the core genes; nodes labels (numbers) correspond to SNP identity numbers in the genotypes data file; arrows indicate the directions of regulatory interactions (activation and repression); note, the specific type of regulatory interaction is not visualized on the graph, though, the details of the activators and repressors subnetworks are depicted in Figures A2 and A3 of Appendix 2.

**Figure A1:**
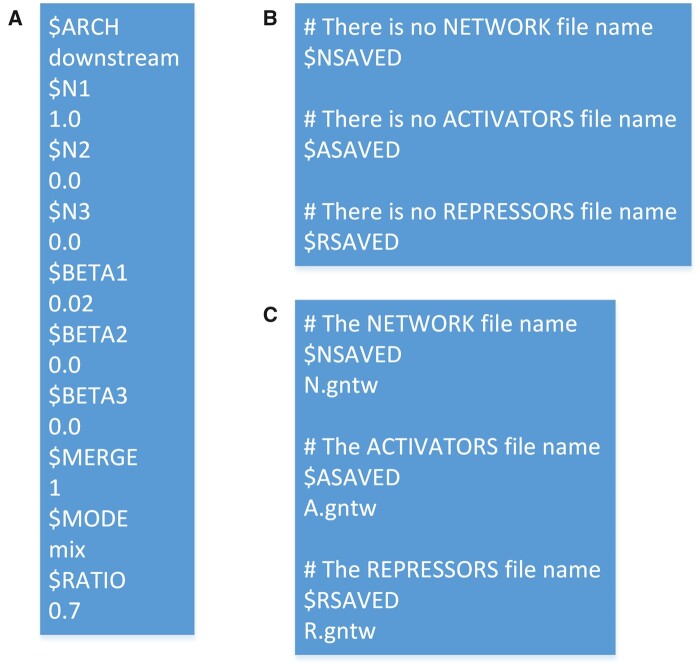
Configuration set-up used in MeSCoT interface in relation to genomic network data. The network options used in (A) *.grm parameters and (B) *.tsim configuration files to produce *in silico* genomic network depicted in Figures 3, A2, and A3; (c) the network options used in *.tsim configuration file to reuse (already simulated) genomic network data.

To demonstrate the MeSCoT functionality of *in silico* networks simulation the software interface (*.grm and *.tsim files in Figure 2A and Figure A1A and B in Appendix 1) was configured such that three data files representing the adjacency matrices N, A, and R (Equations 5 and 6) were generated. The network data were produced once and subsequently reused in all simulation studies. The resulting network geometry represented in terms of a directed graph is depicted in Figure 3.

**Figure 4:**
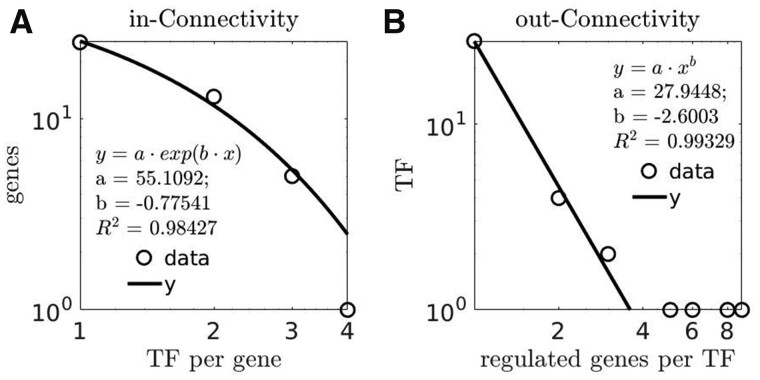
Connectivity properties of simulated regulatory network; (A) log–log plot of in-Connectivity distribution; (B) log–log plot of out-Connectivity distribution; TF stands for transcription factor; dots indicate distribution data; solid lines are distributions’ fit; insets to the plots depict the types and parameters of distributions fitting.

The network was constructed only as a simplicial complex of *1-*dimensional simplexes (where no *2-* and *3-*dimensional simplexes were included in the resulting network, see the Appendix 1 for the details of the software interface configuration), which was sufficient to achieve a basic connectivity properties (Figure 4) typical to genomic regulatory networks (Balaji *et al.* 2006; Van den Bulcke *et al.* 2006; de Matos Simoes *et al.* 2013; Angelin-Bonnet *et al.* 2019; Sarkar *et al.* 2021), including a scale-free distribution characteristic (Strogatz 2001; Broido and Clauset 2019), Figure 4B.

### Expression data

**Figure 5:**
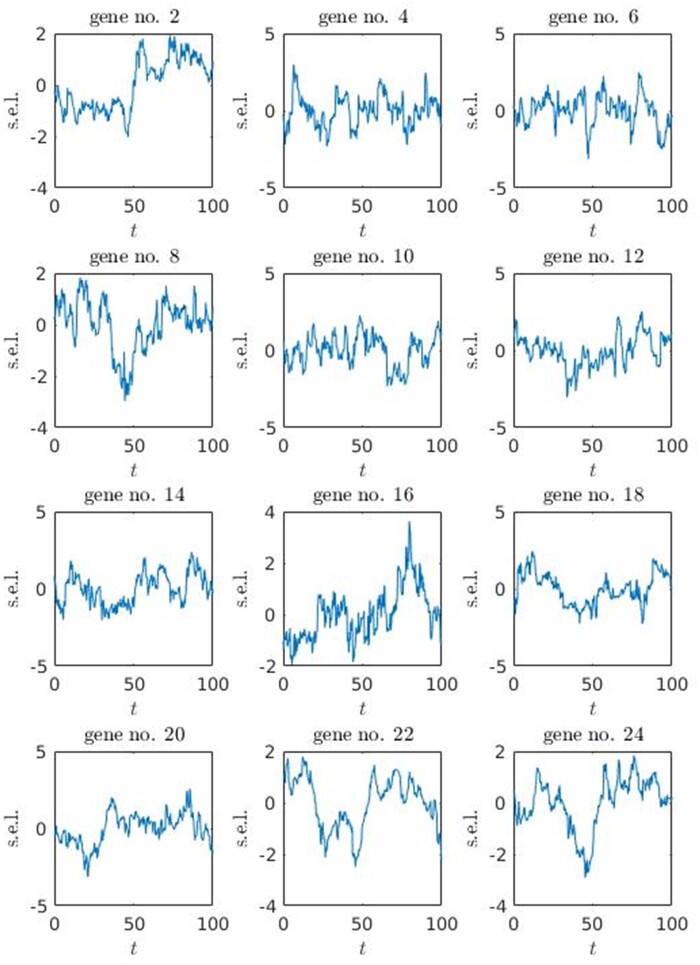
Standardized expression data for core genes. The expression level profiles were generated using mRNA concentration data of reference genotype (see Equation 8 for definition); s.e.l. is standardized expression level, obtained by subtracting mean and dividing by standard deviation; t is time in minutes, the time was adjusted to not include first 20% of dynamic solution in order to avoid the impact of initial condition.

The detailed dynamics of gene regulatory network expressed in terms of mRNA and protein concentrations are covered by basic software functionality. As an example, the standardized expression level profiles of core genes (for the network depicted in Figure 3) are generated using mRNA concentration data and depicted in Figure 5.

### G×G study

**Figure 6:**
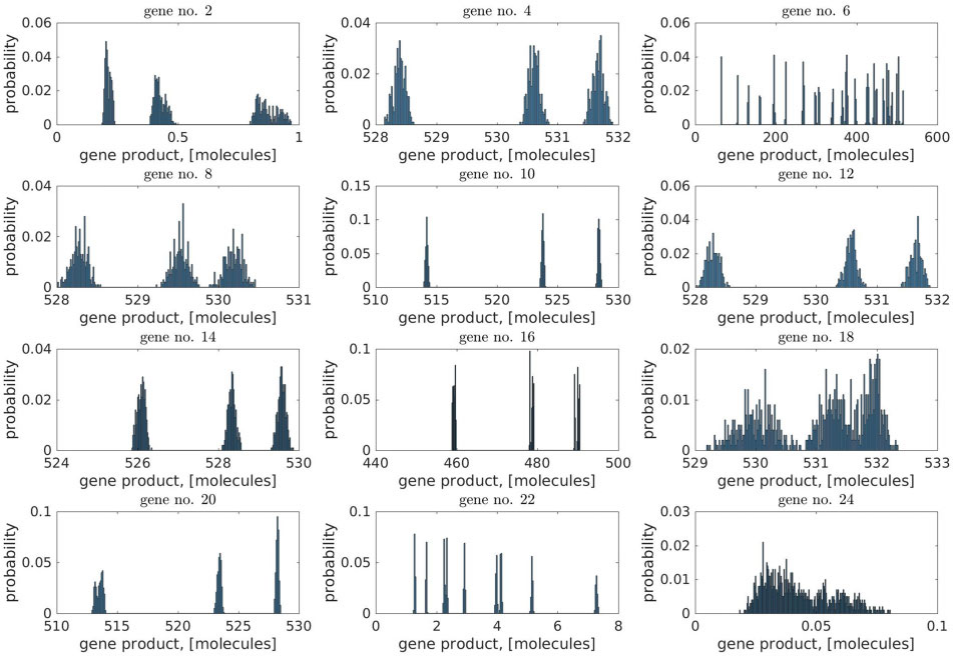
Distributions of core genes’ products. The products represent proteins concentrations [here we use (molecules) though the model allows other units of concentration]; the distributions were generated for the population consisting of 5000 distinct genotypes using *products.txt* file from the software interface (Figure 2B).

The program interface (Figure 2B) allows extensive analysis of G×G interactions. The core genes’ products form complex patterns, such as segregated distributions of protein molecules in Figure 6, due to the interplay between the genomic differences in population and the geometry of the genomic network, Figure 3.

**Figure 7:**
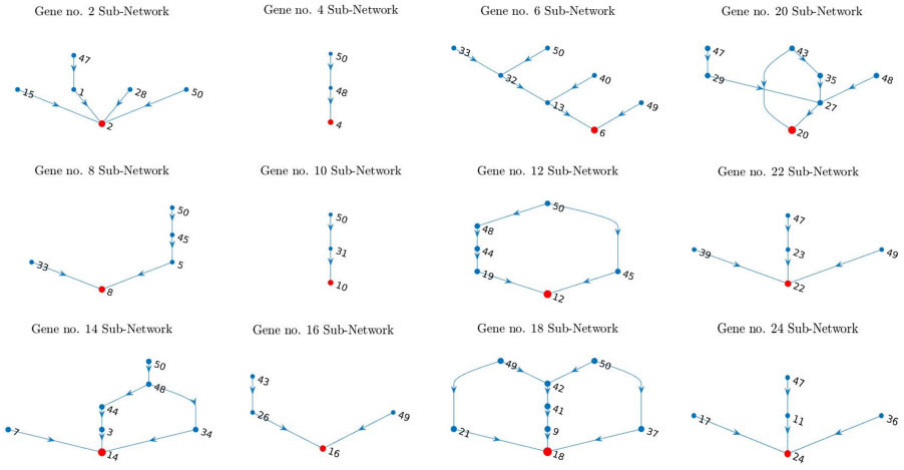
Interaction subnetworks for core genes. The depicted subnetworks associated with the protein distributions in Figure 6; the red nodes indicate the core genes and the blue nodes are the peripheral genes determined in terms of omnigenic model; arrows indicate the directions of regulatory interactions (activation and repression); note, the specific type of regulatory interaction is not visualized on the graph, though, the details of the activators and repressors subnetworks are depicted in Figures A2 and A3 of Appendix 2.

While the effect of genomic variance acts on the genes’ properties related to the regulatory mechanisms (see the model details in the *Methods* section) within the same network for all genotypes, the effect of the network geometry can be conceptualized as the core genes subnetworks interactions. Because it is rather difficult to relate the particular subnetwork geometry depicted in Figure 7 to its product distribution (similar subnetwork geometries correspond to the different products distributions and vice versa, Figure 6), we suppose the subnetworks interaction forms the patterns visualized in Figure 6 (the subnetworks in Figure 7 associated with the protein distributions in Figure 6).

**Figure 8:**
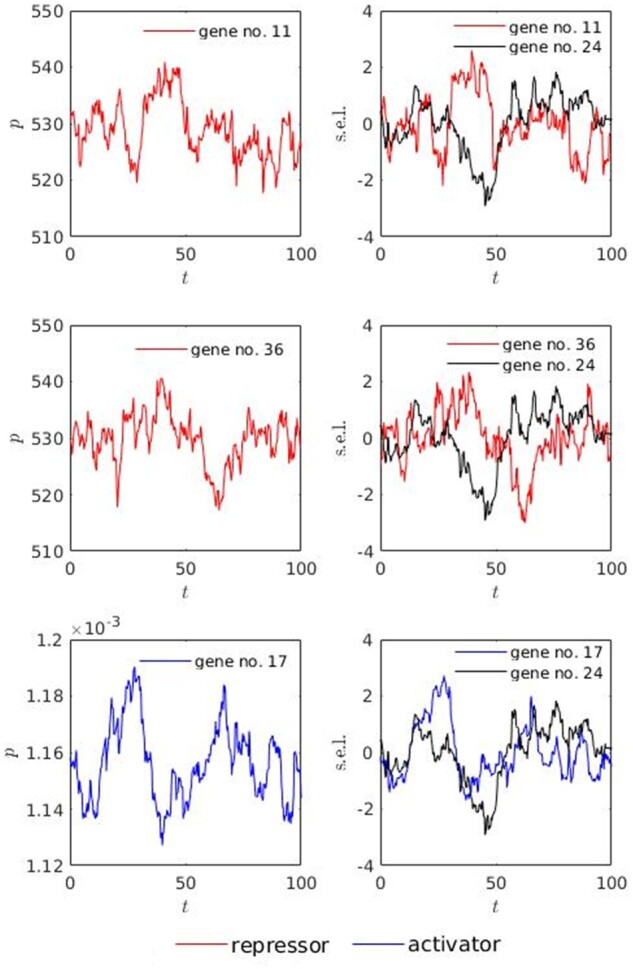
Dynamics of regulatory subnetwork for gene no. 24. The plots at the left side represent protein concentrations for the regulatory genes; the plots at the right side represent the expression level profiles for the regulatory genes; the profiles were generated using mRNA concentration data of reference genotype (see Equation 8 for definition); p is protein concentration [molecules]; s.e.l. is standardized expression level, obtained by subtracting mean and dividing by standard deviation; t is time in minutes, the time was adjusted to not include first 20% of dynamic solution in order to avoid the impact of initial condition; red color represents repressors (gene nos. 11 and 36), blue color represents activator (gene no. 17), black color represents the target gene (gene no. 24).

On a molecular level, the characteristics of protein distributions (the means, in particular) are closely related to genomic interaction within the subnetworks. As an example, the dynamics of regulatory subnetwork for gene no. 24, expressed in terms of its activators and repressors, is depicted in Figure 8. As expected, the s.e.l. values of gene no.24 positively correlate with its activator’s (gene no.17) s.e.l. values and negatively correlate with its repressors’ (genes no. 11 and 36) s.e.l. values (the right column of plots in Figure 8).

**Figure 9:**
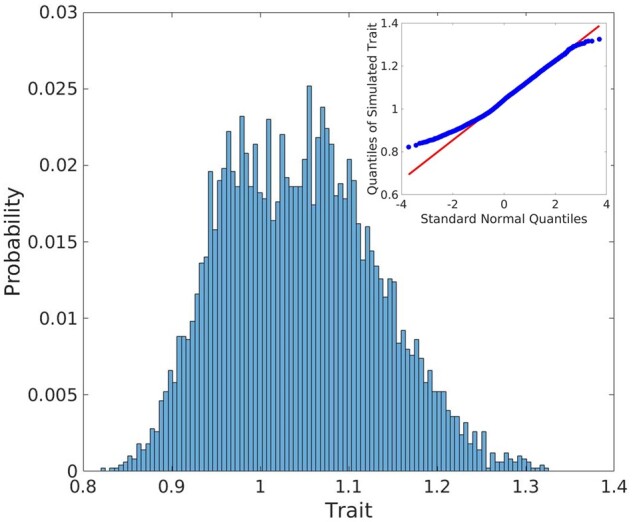
Distribution of simulated genotypic values (traits, expressed as normalized values). The distribution was generated for population consisting of 5000 distinct genotypes using *traits.txt* file from the software interface (Figure 2B); the trait values were calculated using the normalized values of protein concentrations according to Equation 7; the inset to the main plot is the quantile–quantile plot of the genotypic values *vs* standard normal.

Cumulative contribution of all normalized core genes’ products is visualized in Figure 9 where the result is represented as the probability distribution of simulated genotypic values upon 5000 individuals. For the assumed genomic architecture and considered for this study genotypes, the trait distribution tends to be normal, though it is not exactly normal as the inset to the main plot in Figure 9 demonstrates, and appears as a combination of minor distributions related to the individual core genes’ products (Figure 6).

**Figure 10:**
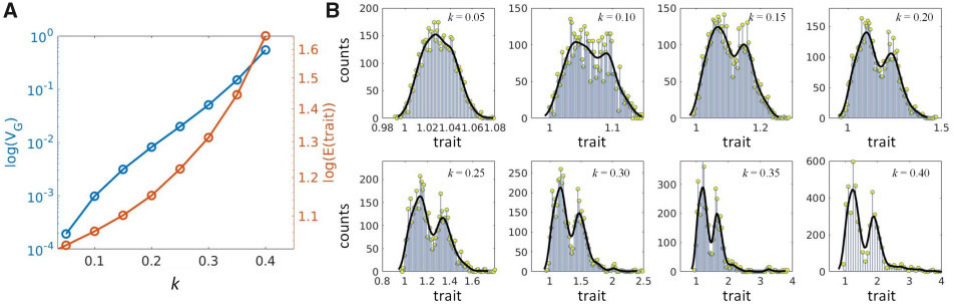
Impact of the G×G response parameter κ on simulated genotypic values (trait). (A) Changes of genomic variance and distribution means in relation to κ; $V_{G}$ is the genomic variance; E(trait) is the genotypic value mean. (B) Segregation of genotypic value distributions in relation to increased values of κ; the distributions appear as a number of counts *vs* genotypic values (trait) which are normalized.

Aside from the architecture-based characteristics of the trait distribution (Figure 9), the response parameter κ plays an additional role in adjustment of the resulting values of genomic variance and determines the compactness of the means of minor distributions that form the trait. Increase of κ magnifies the influence of relative markers effects (Equation 8) resulting in increased values of the genomic variance, Figure 10A (blue line). In addition, it tends to shift the trait distribution mean and increase its segregation, Figure 10A (red line) and Figure 10B. Observed segregation of the trait distribution is right-shifted and it is due to the model solution space is always positive and the trait is normalized but not centered.

Note, as a default, the MeSCoT uses the additive genetic model of quantitative trait (Equation 7). However, the program interface (Figure 2B) allows a custom model of a quantitative trait (here the *products* data files should be utilized) as well a much broad analysis of G×G interactions (here an additional sets of time-series data for each genotype should be considered).

### G×E study

**Figure 11:**
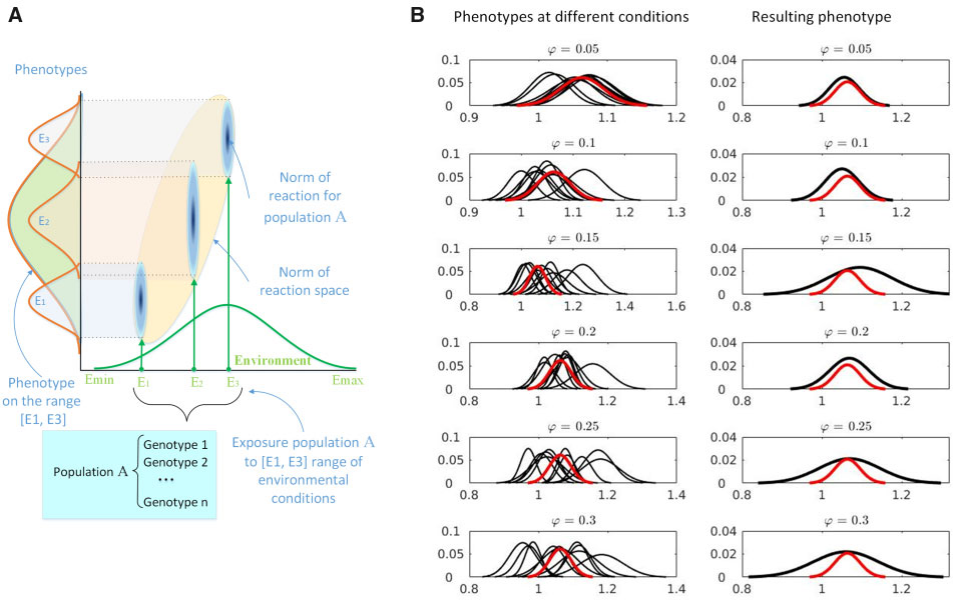
Norm of reaction and the results of G×E study. (A) The concept of the norm of reaction for population. (B) Simulation results of G×E interaction for 6 distinctive environments characterized by φ=0.05 - 0.3; for each environment there are 10 simulated phenotypes (black lines); red line indicates the genotypic value distribution (no environmental impact). Distributions appear as probabilities *vs* normalized phenotypic values.

The G×E study appears as series of G×G simulations with fixed φ. The simulated phenotypes represent the norms of reaction (De Jong 1990; Gomulkiewicz and Kirkpatrick 1992) for the particular environment, Figure 11.

**Figure 12:**
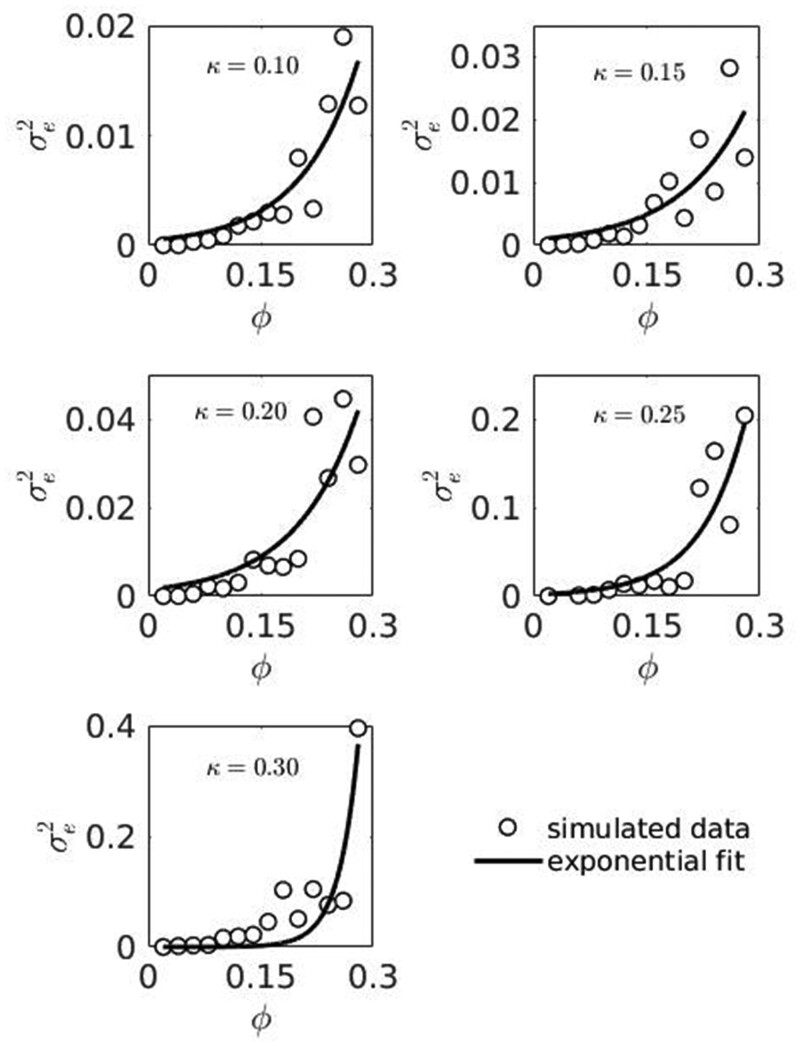
Changes in environmental variance component ${\sigma_{e}}^{2}$ within the phenotypic variance of simulated trait due to φ.

G×E study reveals exponential increase of values of environmental variance component in response to increased parameter φ, Figure 12.

### Performance tests

**Figure 13:**
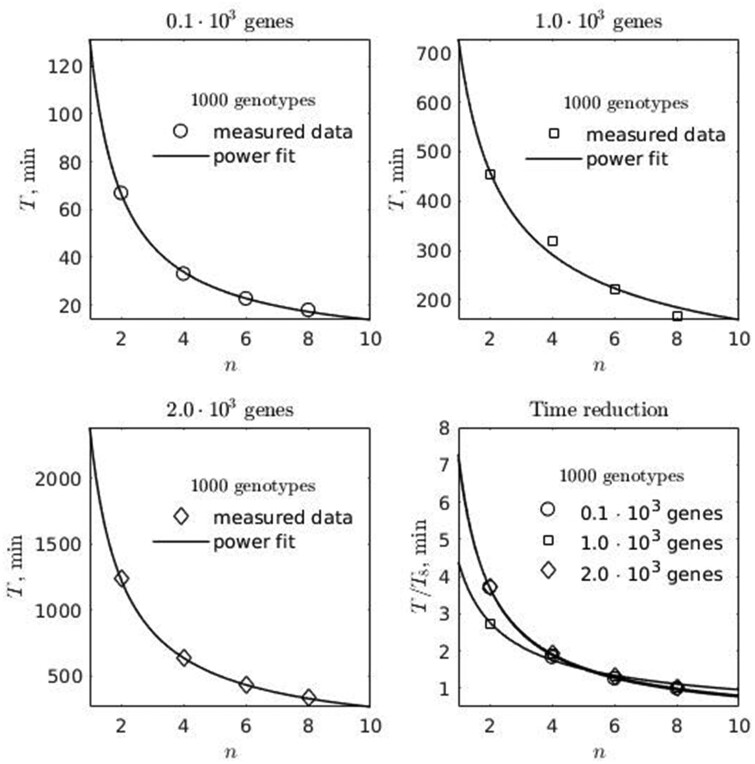
The results of MeSCoT performance tests. The tests were conducted on HPC cluster of Intel servers of Skylake architecture; the requested memory was limited to 50 GiB (though never has reached this value), the maximum observed CPU frequency during the tests was 3.5 GHz and the number of requested computing threads 2–8; all the tests were conducted on 1000 genotypes data with variable size of genomic network where the number of genes involved was 100, 1000, 2000; T is an elapsed time; n is number of computing threads; $T_{8}$ is the elapsed time of computations where 8 threads were involved.

The results of high-performance computing (HPC) tests, which demonstrate the computing time and scalability of the software, are depicted in Figure 13. Because the software multithreading functionality is implemented such that blocks of multiple genotypes belong to separate threads, all HPC tests were conducted using 1000 genotypes data while the number of SNP variants (genes involved in regulatory network) was variable.

**Figure 14:**
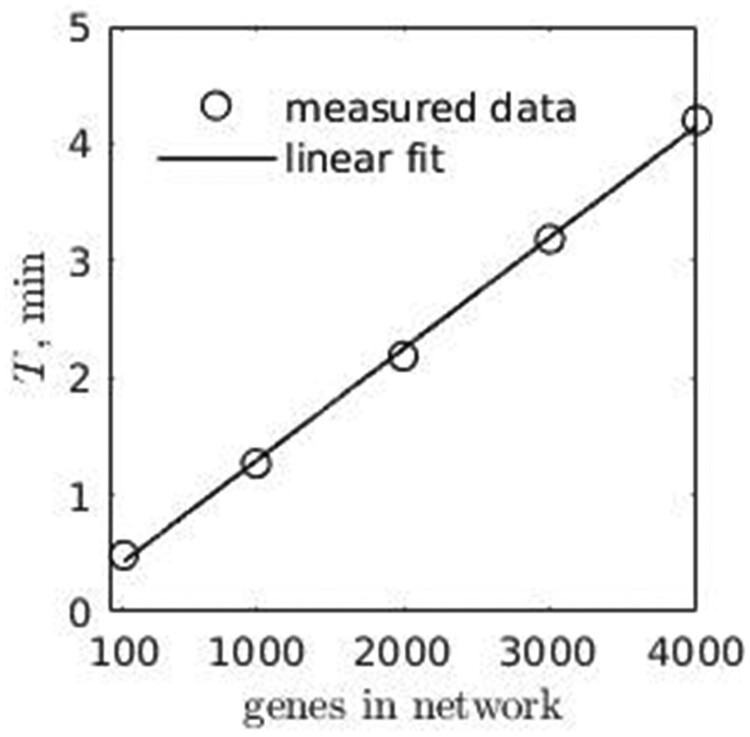
Computing time for reference genotype (1-threaded process). The tests were conducted on HPC cluster of Intel servers of Skylake architecture; the requested memory was limited to 10 GiB, the maximum observed CPU frequency during the tests was 3.5 GHz and the number of computing threads 1; T is an elapsed time.

The estimated time reduction coefficient for 8-threaded process was ∼0.125 compare to 1-threaded process, Figure 13 (Time reduction plot). The time required to complete one genotype calculation shows linear scaling in relation to a number of genes in regulatory network, Figure 14.

## Figures and Tables

**Figure A2:**
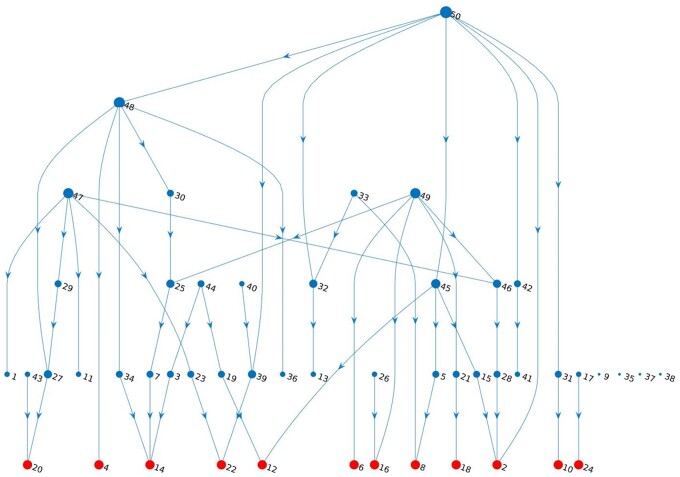
The simulated activators genomic subnetwork. The depicted subnetwork represents the adjacency matrix A∈N (Equation 5) and corresponds to the network depicted in Figure 3; the arrows indicate the directions of genes activation regulation.

**Figure A3:**
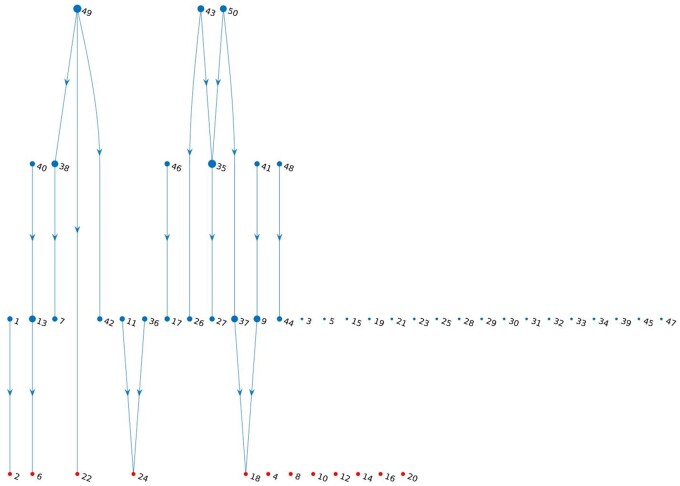
The simulated repressors genomic subnetwork. The depicted subnetwork represents the adjacency matrix R∈N (Equation 5) and corresponds to the network depicted in Figure 3; the arrows indicate the directions of genes repression regulation.

## Appendix 1: The network geometry

The details of configuration set-up used in MeSCoT interface in relation to genomic network data are depicted in Figure A1. The simulated activators genomic subnetwork and repressors genomic subnetwork are shown in Figures A2 and A3, respectively.

## Appendix 2: Model parameters

**Table A1:** Nomenclature of the model variables/parameters and their corresponding software implementation

Description	Symbol	Software			
		Keyword	Default value	Default unit	Allowed interval
mRNA concentration	X	Non	Calculated	[a.unit]	[0,+inf)
Protein concentration	S	Non	Calculated	[a.unit]	[0,+inf)
Binding probabilities of RNAP II to a promoter region of a gene	P	Non	Calculated	Non	[0,1]
Time	t	$TMAX	500	[min]	[0,+inf)
Time delay	τ	$TDIL	0	[min]	[0,+inf)
Rate constant of mRNA transcription	$K_{x}$	$RATERNA	4.0	[1/min]	[0,+inf)
Rate constant of protein translation	$K_{s}$	$RATEP	8.0	[1/min]	[0,+inf)
Degradation constant of mRNA	$Z_{x}$	$DEGRNA	0.2	[1/min]	[0,+inf)
Degradation constant of protein	$Z_{s}$	$DEGP	0.6	[1/min]	[0,+inf)
Stochastic matrix variance	${\sigma_{q}}^{2}$	$STOCH	0.0	Non	[0,1]
Stochastic matrix constant	γ	Non	Predefined	Non	Non
Number of genes in the network	n	$SNP	Non	Non	[0,+inf)
Relative free energy related to a gene’s RNAP II binding	$G_{p}$	$EBIND	9.0	[kT]	[0,11]
RNAP II binding constant	H	$KBIND	8.1e3	[1/kT]	[0,+inf)
Gene’s regulatory factor	F(s)	Non	Calculated	Non	[0,+inf)
Gene’s activator factor	${{F(s)}_{A}}_{i}$	Non	Calculated	Non	[0,+inf)
Gene’s repressor factor	${{F(s)}_{R}}_{i}$	Non	Calculated	Non	[0,+inf)
Concentration of activator	$S_{\mathrm{Ai}}$	Non	Calculated	[a.unit]	[0,+inf)
Concentration of repressor	$S_{\mathrm{Ri}}$	Non	Calculated	[a.unit]	[0,+inf)
Relative free energy related to a gene’s activator binding	$G_{A}$	$EACT	6.0	[kT]	[0,11]
Relative free energy related to a gene’s repressor binding	$G_{R}$	$EREP	7.0	[kT]	[0,11]
Activator binding interaction constant	Φ	$KACT	5.0e5	[1/kT]	[0,+inf)
Activator constant	$h_{A}$				
Repressor constant	$h_{R}$				
Adjacency matrix of activator subnetwork	A	$ASAVED	Calculated	Non	Non
Adjacency matrix of repressor subnetwork	R	$RSAVED	Calculated	Non	Non
Adjacency matrix of the genomic network	N	$NSAVED	Calculated	Non	Non
Genotypic value	y	non	Calculated	[n.value]	[0,+inf)
Matrix of weights	W	$CORE	Non	Non	[0,1]
Number of core genes	$n_{c}$	$CORE	Non	Non	[0,+inf)
Number of individuals in population	m	$SNP	Non	Non	[1,+inf)
Time averaged and normalized value of core gene’s protein	${\overset{-}{S}}_{\mathrm{ji}}$	Non	Calculated	[a.unit]	[0,+inf)
Matrix of relative markers’ effects	M	Non	Calculated	non	non
Matrix of reference genotypes	$M_{\mathrm{ref}}$	Non	Calculated	non	non
Genotypic matrix	$M_{\mathrm{pop}}$	$SNP	Non	non	non
G×G response paramete	κ	$SNPDIFF	0.25	non	[0,1]
G×E response parameter	Φ	$GENDIFF	0.0	non	[0,1]

[a.unit] means arbitrary (user defined) units can be used in the model; [n.value] means normalized value.
